# Applicability of anthropometric indices for assessing speed and agility in children and adolescents: proposed reference values by age and sex

**DOI:** 10.1016/j.jped.2026.101596

**Published:** 2026-09-05

**Authors:** Marco Cossio-Bolaños, Ruben Vidal-Espinoza, Mileny Caroline Menezes de Freitas, Camilo Urra-Albornoz, Fernando Alvear-Vasquez, Luis Felipe Castelli Correia de Campos, Jose Sulla-Torres, Rossana Gomez-Campos

**Affiliations:** a Universidad Católica del Maule, Talca, Chile; b Universidad Católica Silva Henriquez, Santiago, Chile; c Universidade Estadual de Londrina, Londrina, PR, Brazil; d Universidad del Bío Bío, Chillán, Chile; e Universidad Santo Tomás, Chile; f Universidad Adventista de Chile, Chillán, Chile; g Universidad Católica de Santa María, Arequipa, Peru; h Universidade Estadual de Campinas, Campinas, SP, Brazil

**Keywords:** Anthropometric índices, Predictors, Velocity, Agility, Percentiles, Schoolchildren

## Abstract

**Objective:**

a) To verify the applicability of anthropometric indices (body mass index (BMI), tri- weight index (TWI), body surface area (BSA)) for evaluating velocity and agility tests in children and adolescents, and to propose reference values.

**Methods:**

A descriptive cross-sectional (correlational) study was designed in 1573 schoolchildren aged 6 to 17 years. Weight and height were assessed. BMI, TWI, and BSA were calculated. The 20-meter sprint and agility (5 meters x 10 repititions) test was evaluated.

**Results:**

In the individual analysis, the explanatory power for velocity and agility for BMI was almost nil. In the case of the TWI (r² = 0.123 and 0.110) and BSA (r² = 0.157 and 0.211), they showed significant explanatory power. When age was incorporated, the predictive power significantly increased. For BMI+age (r² = 0.342 and 0.435); TWI+age (r² = 0.382 and 0.467), and BSA+age (r² = 0.292 and 0.394). When sex was added, the results showed a further improvement. For BMI+age+sex (r² = 0.409 and 0.498); TWI+age+sex (speed test (r² = 0.435 and 0.517) and BSA+age+ sex (r² = 0.373 and 0.470).

**Conclusion:**

The TWI better explained motor performance than the BMI and BSA in Chilean schoolchildren. Models that included TWI along with age and sex indicated that the TWI could be an alternative for assessing velocity and agility. Furthermore, percentiles were developed for the 20-meter sprint and the 5-meter agility test (10 repetitions), which are useful for monitoring and interpreting changes in the physical performance of schoolchildren.

## Introduction

Anthropometric indices can be used in pediatric populations to assess the general health status, nutritional adequacy, and growth and development patterns of children and adolescents [1]. These indices are often used in school systems in various regions of the world to assess weight status (identifying the prevalence of underweight, normal weight, and obesity)[2].

Anthropometric indices are generally derived from two classic, raw measurements (e.g., weight and height) and can be interpreted individually and collectively. Among the best-known indicators are body mass index (BMI), tri-weight index (TWI), and, more recently, body surface area (BSA), which is considered a better predictor of body size and weight status in children, adolescents, and adults [3].

In recent years, several studies have used BMI as a predictor of physical fitness in children and young people, obtaining moderate correlations with physical tests of velocity and agility [4,5]. TWI has even been considered an excellent predictor of physical performance in children and adolescents, surpassing BMI [6]. However, a recent study conducted on Peruvian schoolchildren has described that BSA surpasses BMI and TWI in analyzing the physical fitness of schoolchildren [7].

In this regard, studying the relationships between BMI, TWI, and BSA with velocity and agility tests in Chilean schoolchildren is extremely relevant. Both physical tests are part of the motor component of physical fitness, the contents of which are evaluated and developed in physical education classes during childhood and adolescence.

In fact, some studies highlight that, for example, velocity, agility without a ball, and vertical jump ability share common physiological and biomechanical determinants [8]. Furthermore, strength and coordination, as well as velocity and agility, are closely related to each other, as they interact continuously in the performance of motor acts and, therefore, should be considered as a single unit [9].

In fact, considering that sprint velocity and agility are physical abilities that depend on the interaction between the musculoskeletal and neuromotor systems [10,11], anthropometric indices based on weight and height are likely related to body size and proportionality. Therefore, these indicators could play a relevant role in the motor performance of these abilities.

To this end, the need arises to identify which of these anthropometric indicators might best explain the motor performance of velocity and agility tests in Chilean schoolchildren.

Therefore, the initial objective of this study was to verify the applicability of anthropometric indices (BMI, TWI, and BSA) for evaluating physical tests of velocity and agility in Chilean children and adolescents. Furthermore, it aimed to propose reference values ​​according to age and sex.

## Materials and methods

### Type of study and sample

A descriptive cross-sectional (correlational) study was designed involving 1573 schoolchildren (873 males and 697 females) aged 6 to 17 years. The sampling used in this study was non-probabilistic (accidental). All schoolchildren in the study belong to eight municipal schools in the Maule region (Chile). This region has four provinces (Curicó, Cauquenes Talca, and Linares). Two schools were evaluated in each province, totaling eight schools in the urban area of the region.

One of the researchers contacted the principals of each school to explain and describe the objective of the project. Once each school agreed to participate, a meeting was arranged with the parents and/or guardians of the schoolchildren to obtain their authorization for their children to participate. A consent form was sent to them so that they could accept or reject the participation of their minor children.

**Figure 1:**
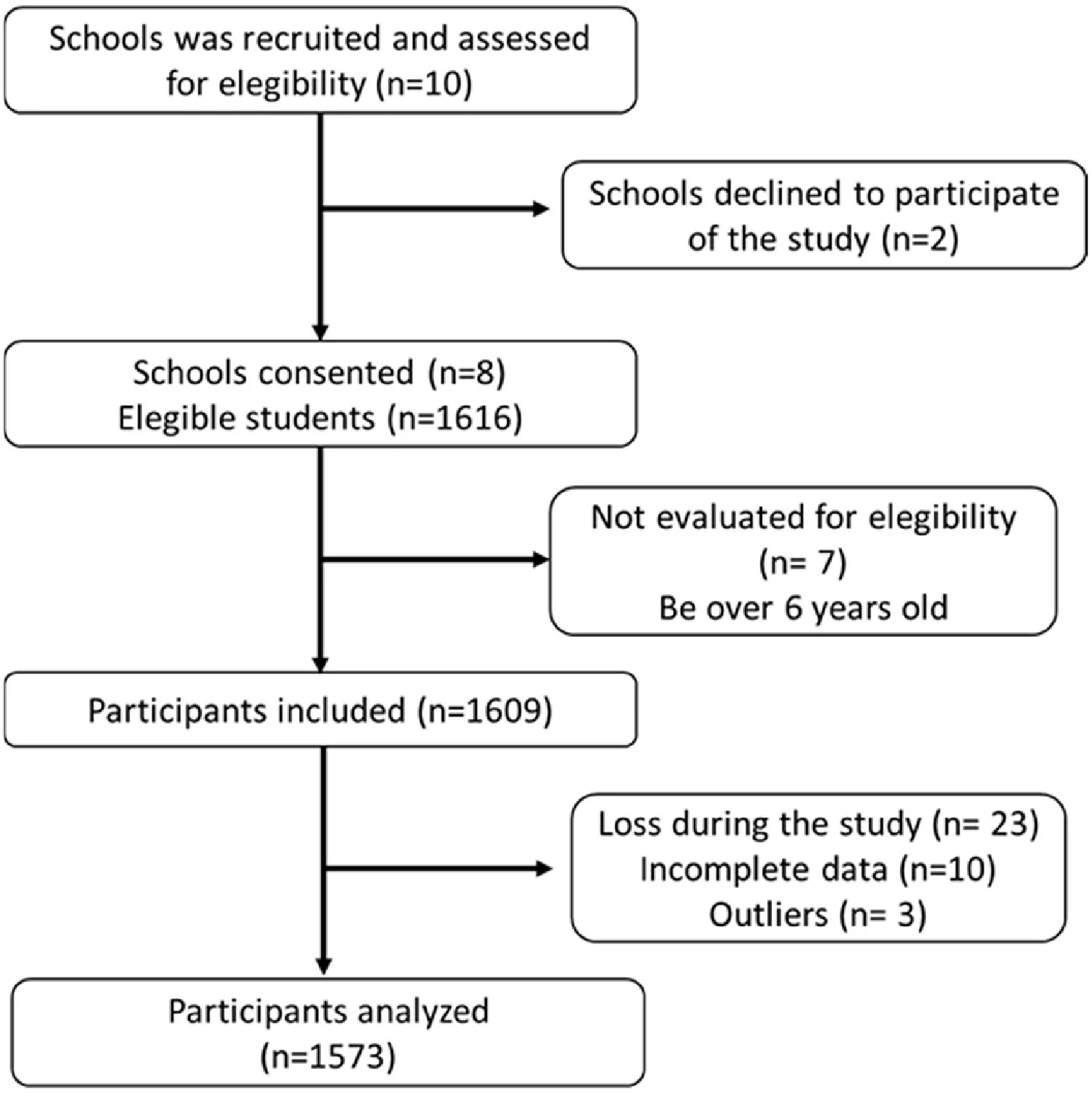
Flowchart of the study sample selection process. Source: the authors.

The study included schoolchildren who were within the established age range (6 to 17 years) and who completed the anthropometric measurements and physical tests. Those who did not attend on the days of the evaluation, those who did not complete the tests, and those who were on leave or medical leave were excluded (Figure. 1).

The entire evaluation process was carried out in accordance with the Declaration of Helsinki for human subjects and the recommendations of the Ethics Committee of the Autonomous University of Chile (UA 238–2018).

### Techniques and instruments

Anthropometric assessments and physical tests (velocity and agility) were conducted at each school's facilities. To this end, a team of four physical education professionals with extensive experience in anthropometric and physical measurements was established. The assessments were carried out between April and June 2023. A space was set up at each school for anthropometric measurements and physical tests.

The protocol described by Ross and Marfell-Jones [12] was used for the anthropometric assessment of weight and height. The assessments were carried out with as little clothing as possible (barefoot, shorts, and T-shirt). For example, an electronic scale (Tanita BC 730, UK) with an accuracy of 100 g and a range of 0 to 150 kg was used to measure weight. Standing height was measured using a portable stadiometer (Seca 216, Gmbh & Co. KG, Hamburg, Germany) with an accuracy of 1 mm.

Weight and height for each age and sex were used to calculate anthropometric indicators. The indices determined were: Body Mass Index [BMI = weight (kg) / height 2 (m)], Tri-Weight Index [TWI = weight (kg) / height 3 (m)] and Body Surface Area [BSA = 0.007,184 × weight (kg)0.425 × height (cm)0.725, using the Du Bois formula [13].

Maturity status was determined using a non-invasive anthropometric method developed by Moore et al.​ [14]. for both sexes. These equations use data such as chronological age, standing height, and constants. The results obtained express values (positive and/or negative), which are interpreted as years before or after the Peak Growth Velocity (APHV). The equations provide results at the following levels: −5, −4, −3, −2, −2, −1, 0, 0, +1, +1, +2, +3, +4, +5 APHV. For example, zero (0) represents the time of the APHV, negative values represent the years remaining to reach the PHV, and positive values represent the years elapsed after the PHV.

The physical tests were evaluated after a 10-minute warm-up (warm-up, jogging, flexibility exercises). A sequence was then organized to initially evaluate the 20-meter sprint test, followed by the agility test (5 m × 10 repetitions). Both tests were performed on a hard surface (basketball court).

The 20 m sprint test was evaluated according to the suggestions described by Grosser & Starischka [15]. The course was marked out with three cones (for example, a starting cone, a second cone 20 m away, and a third cone 25 m away). The subject is placed in a high starting position, then at the evaluator's signal (ready, go) the stopwatch is pressed to activate it, then when the subject passes the 20 m mark, it is pressed again to record the time in seconds. The person being evaluated must continue running until they complete the 25 m This procedure was evaluated twice (with an interval of 3 to 5 min between each test), recording the best time. A Casio® 1/100 (*sec*) stopwatch was used.

For the agility test (5 m × 10 reps), the distance of 5 m is marked with adhesive tape, as described by Verschuren et al.​ [16]. The subject stood at one end and, at the evaluator's command (ready, set, go), ran at maximum velocity from one end to the other, repeating the action 10 times (back and forth). The test ended where it began. A Casio® 1/100 (*sec*) stopwatch was used for this purpose. This action was repeated twice, with a 3- to 5-minute rest interval, and the best time was recorded.

The reliability of both physical tests was established through test-retest reliability. The intra-rater relative technical measurement error values ​​ranged from 1.1% to 1.3%.

### Statistics

The normal distribution of the data was verified using the Kolmogorov-Smirnov test. Descriptive statistics were calculated using the arithmetic mean and standard deviation. Differences between the two sexes in each age group were verified using the t-test for independent samples.

To examine the explanatory power of anthropometric indices (BMI, TWI, and BSA) on physical tests (speed and agility), a hierarchical multiple linear regression analysis was performed. Successive models were generated for each index: a) an unadjusted model including only the anthropometric index; b) a model adjusted for age; and c) a model adjusted for age and sex. The predictive power of each model was evaluated considering the multiple correlation coefficient (r), the coefficient of determination (r²), the adjusted coefficient of determination (adjusted r²), the standard error of the estimate (SEE), and the change in explained variance (ΔR²) between consecutive models. The independent contribution of each variable was estimated using standardized beta coefficients (β). Additionally, the absence of multicollinearity problems was verified using the tolerance index and the variance inflation factor (VIF). These were considered acceptable.

The data were processed and calculated in Excel, SPSS 16.0, and Medcalc v.23.4. LMS ChartMaker version 2.316 [17] software was used to develop the reference values. The percentile curves were smoothed to create three specific curves by age and sex according to Cole et al. [18], for example, L (lambda; asymmetry), M (mu; median), and S (sigma; coefficient of variation). The percentiles (P3, P5, P10, P15, P25, P50, P75, P85, P90, P95, and P97) were calculated. A significance level of 0.05 was adopted in all case.

## Results

Table 1 shows the values of anthropometric indices (BMI, TWI, and BSA) and physical tests of velocity and agility in Chilean schoolchildren by age and sex. There were no significant differences in body weight between the sexes from ages 6 to 12 (p > 0.05). However, from age 13 to 17, males had higher body weight than their female counterparts (p < 0.001). In terms of height, both sexes had similar values from ages 6 to 12 (p > 0.001), and from age 13 onwards, males had significantly higher values than females up to age 17 (p < 0.001). In terms of BMI, there were no differences between the sexes at early ages up to 10 years old, but from 11 to 15 years old, males had higher BMI values than females (p < 0.001). Similar results were obtained for TWI, with differences observed from ages 11 to 15 (p < 0.001), where males had higher values, and similar results at ages 16 and 17 (p > 0.001). In BSA, the differences are very marked during adolescence, with males having higher values from ages 14 to 17 (p < 0.001). Meanwhile, at younger ages, there were no differences (p > 0.001), with values being similar in both sexes.

**Table 1:** Anthropometric and physical characteristics of the schoolchildren studied by age and sex.

Age (years)	n	Weight (kg)		Height (cm)		BMI (kg/m^2^)		TWI (kg/m^3^)		BSA (cm^2^)		Velocity 20 m (seg)		Agility (seg)	
		X	SD	X	SD	X	SD	X	SD	X	SD	X	SD	X	SD
**Males**															
6	45	27.19	4.68	121.53	5.61	18.31	2.18	15.08	1.73	0.95	0.09	5.77	0.72	28.27	4.97
7	53	29.58	5.89	125.59	5.29	18.63	2.64	14.82	1.88	1.00	0.11	5.50	0.93	26.19	4.01
8	70	33.99	7.97	131.69	7.97	19.45	3.43	14.81	2.82	1.10	0.15	5.10	0.67	24.44*	3.76
9	70	37.76	8.81	139.02	9.52	19.44	3.74	14.03	2.62	1.18	0.21	4.86	0.7	22.81*	2.84
10	76	42.52	10.74	143.7	7.77	20.41	3.97	14.20	2.63	1.29	0.18	4.69	0.83	21.67*	2.42
11	80	46.62	10.06	149.96	7.68	20.16*	3.60	13.45*	2.37	1.37	0.16	4.33*	0.65	20.43*	2.32
12	75	52.44	11.52	158.53*	8.44	20.70*	3.40	13.06*	2.03	1.52	0.18	4.06*	0.59	19.05*	2.48
13	89	59.19*	12.74	165.20*	7.92	21.53*	3.54	13.03*	2.02	1.64	0.19	3.98*	0.5	18.86*	1.56
14	84	62.00*	11.69	168.99*	6.71	21.63*	3.33	12.80*	1.93	1.71*	0.17	3.78*	0.46	17.69*	1.89
15	99	66.62*	11.52	171.12*	6.37	22.71*	3.50	13.29*	2.08	1.78*	0.16	3.87*	0.37	18.33*	1.81
16	94	70.02*	16.2	170.93*	6.55	23.89	4.99	13.98*	2.9	1.79*	0.27	3.96*	0.51	18.15*	2.07
17	41	76.01*	17.12	175.00*	6.62	24.74	5.05	14.14	2.86	1.91*	0.20	3.78*	0.38	18.39*	2.57
Total	876	52.93	17.62	156.05	17.36	21.13	3.96	13.6	2.36	1.50	0.33	4.30	0.83	20.23	3.79
**Females**															
6	48	25.52	4.92	120.63	5.41	17.50	2.92	14.54	2.51	0.92	0.09	5.76	0.59	28.32	3.85
7	68	31.43	8.79	127.24	9.44	19.23	4.05	15.17	3.29	1.04	0.16	5.45	0.73	26.65	3.36
8	57	33.98	7.83	132.49	6.14	19.25	3.57	14.53	2.63	1.11	0.13	5.24	0.89	25.33	2.67
9	60	40.18	10.58	139.82	7.14	20.37	4.41	14.57	3.13	1.23	0.17	5.13	0.79	23.93	3.62
10	55	42.23	11.22	145.42	7.50	19.71	3.78	13.52	2.28	1.27	0.25	4.82	0.64	22.35	2.3
11	57	49.54	13.14	151.03	6.15	21.61	5.1	14.3	3.29	1.43	0.17	4.83	0.57	22.29	1.87
12	58	55.21	9.63	154.42	6.39	23.19	4.07	15.07	2.87	1.52	0.13	4.63	0.59	21.87	2.46
13	50	55.18	8.38	157.98	5.90	22.13	3.42	14.05	2.39	1.52	0.25	4.51	0.63	20.68	2.33
14	72	59.53	14.03	158.68	6.49	23.53	4.73	14.83	2.94	1.60	0.19	4.51	0.72	21.20	2.74
15	71	62.13	14.58	159.69	6.52	24.30	5.25	15.23	3.28	1.64	0.18	4.66	0.7	20.99	2.8
16	62	61.94	10.66	159.71	5.05	23.91	5.07	14.99	3.26	1.61	0.24	4.62	0.55	20.91	2.68
17	39	60.64	10.78	159.88	7.08	23.66	3.38	14.82	2.16	1.62	0.16	4.78	0.95	20.83	2.33
Total	697	48.65	16.41	147.72	14.8	21.65	4.76	14.67	2.92	1.39	0.30	4.90	0.79	22.84	3.70

Legend: X, Mean; SD, Standard deviation; BMI, Body Mass Index; TPI, Tri-Weight Index; BSA, Body Surface Area; Sec: Seconds; *, Significant difference compared to women (p < 0.05).

Table 2, Table 3 describe the comparisons of linear regression models used to explain the 20-meter sprint and the agility test (5 m x 10 repetitions), based on anthropometric indicators in children and adolescents of both sexes.

**Table 2:** Linear regression models to explain the relationship with the 20-meter sprint test with anthropometric indices in schoolchildren of both sexes.

Model	R	R²	Adj R²	SEE	ΔR²		β	Sig.	Tol	VIF
	**Velocity**									
BMI	.004	0.000	−0.001	0.865	0.000	Constant		0.000		
						β BMI	−0.004	0.868	1.000	1.000
BMI + Age	.585	0.342	0.341	0.702	0.342	Constant		0.000		
						β BMI	0.244	0.000	0.847	1.180
						β Age	−0.635	0.000	0.847	1180
BMI+Age+Sex	.640	0.409	0.408	0.666	0.067	Constant		0.000		
						β BMI	0.217	0.000	0.839	1.191
						β Age	−0.600	0.000	0.834	1.199
						β Sex	0.262	0.000	0.982	1.018
TWI	.351	0.123	0.123	0.810	0.123	Constant		0.000		
						β TWI	0.351	0.000	1.000	1.000
TWI + Age	.618	0.382	0.381	0.681	0.258	Constant		0.000		
						β TWI	0.302	0.000	0.991	1.009
						β Age	−0.511	0.000	0.991	1.009
TWI+Age+Sex	.660	0.435	0.434	0.651	0.053	Constant		0.000		
						β TWI	0.261	0.000	0.960	1.042
						β Age	−0.493	0.000	0.985	1.015
						β Sex	0.235	0.000	0.961	1.041
BSA	.396	0.157	0.157	0.795	0.157	Constant		0.000		
						β BSA	−0.396	0.000	1.000	1.000
BSA + Age	.541	0.292	0.291	0.728	0.135	Constant		0.000		
						β BSA	0.048	0.139	0.406	2.461
						β Age	−0.577	0.000	0.406	2.461
BSA+Age+Sex	.610	0.373	0.371	0.686	0.080	Constant		0.000		
						β BSA	0.083	0007	0.404	2.476
						β Age	−0.577	0.000	0.406	2.461
						β Sex	0.286	0.000	0.985	1.015

Legend: R, correlation coefficient; R^2^, the coefficient of determination; Adj R, adjusted coefficient of determination; SEE, standard error of the estimate; ΔR², change in explained variance; β, beta coefficients; VIF, variance inflation factor; Tol, tolerance index.

**Table 3:** Linear regression models to explain the relationship with the agility test (5 m × 10 repetitions) with anthropometric indices in schoolchildren of both sexes.

Model	R	R²	Adj R²	SEE	ΔR²		β	Sig.	Tol	VIF
	**Agility**									
BMI	.055	0.003	0.002	3.962	0.003	Constant		0.000		
						β BMI	−0.055	0.020	1000	1.000
BMI + Age	.659	0.435	0.434	2.985	0.431	Constant		0.000		
						β BMI	0.220	0.000	0.851	1.176
						β Age	−0.712	0.000	0.851	1.176
BMI+Age+Sex	.706	0.498	0.497	2.814	0.063	Constant		0.000		
						β BMI	0.192	0.000	0.842	1.188
						β Age	−0.681	0.000	0.840	1.191
						β Sex	0.254	0.000	0.983	1.017
TWI	.332	0.110	0.110	3.743	0.110	Constant		0.000		
						β TWI	0.332	0.000	1.000	1.000
TWI + Age	.683	0.467	0.466	2.898	0.356	Constant		0.000		
						β TWI	0.273	0.000	0.990	1.010
						β Age	−0.600	0.000	0.990	1.010
TWI+Age+Sex	.719^c^	0.517	0.516	2.760	0.050	Constant		0.000		
						β TWI	0.229	0.000	0.955	1.047
						β Age	−0.586	0.000	0.986	1.014
						β Sex	0.228	0.000	0.958	1.044
BSA	.460	0.211	0.211	3.524	0.211	Constant		0.000		
						β BSA	−0.459	0.000	1.000	1.000
BSA+Age	.628	0.394	0.394	3.089	0.183	Constant		0.000		
						β BSA	0.052	0.069	0.412	2.429
						β Age	−0.667	0.000	0.412	2.429
BSA+Age+Sex	.686	0.470	0.469	2.891	0.076	Constant		0.000		
						β BSA	0.091	0.001	0.408	2.449
						β Age	−0.675	0.000	0.411	2.431
						β Sex	0.277	0.000	0.986	1.014

Legend: R, correlation coefficient; R^2^, the coefficient of determination; Adj R, adjusted coefficient of determination; SEE, standard error of the estimate; ΔR², change in explained variance; β, beta coefficients; VIF, variance inflation factor; Tol, tolerance index.

Regarding the sprint test, the anthropometric indicators were analyzed individually, according to age and sex. For example, in the individual analysis, the BMI showed no explanatory power for sprint (R² = 0.000; β = −0.004; p = 0.868). However, the TWI (R² = 0.123; β = 0.351; p < 0.001) and the BSA (R² = 0.157; β = −0.396; p < 0.001) showed significant explanatory power. On the other hand, when age was incorporated into the three anthropometric indicators, the predictive power increased significantly. For TWI + age (R² = 0.382, β TWI = 0.302, β age = −0.511, p = 0.000). For BMI + age (R² = 0.342, β BMI = 0.244, β Age = −0.635, p = 0.000). And for BSA + age (R² = 0.292; β BSA = 0.048, β Age = −0.577, p = 0.139). Furthermore, when sex was added, the results showed an additional improvement in all three models. For the model with TWI + age + sex, the results indicate that it showed slightly better fit in explaining the velocity test (R² = 0.435; β TWI = 0.261, β Age = −0.493, β Sex = 0.235; p = 0.000). This was followed by BMI + age + sex (R² = 0.409; β BMI = 0.217, β Age = −0.600; β sex = 0.262, p = 0.000). And BSA + age + sex (R² = 0.373; β BSA = 0.083, β age = −0.577, β Sex = 0.286, p = 0.000). In summary, age was the most important predictor in all models (β = −0.493). Furthermore, the TWI showed a positive and independent contribution (β = 0.261). Sex also contributed significantly (β = 0.235).

Regarding the agility test, the results in Table 3 indicate that, through individual analysis, the BMI showed almost no explanation of the variance (R² = 0.003), although it was statistically significant (β = −0.055, p = 0.020). Meanwhile, the TWI showed moderate explanatory power (R² = 0.110, β = 0.332, p < 0.001). However, the BSA presented the greatest explanatory power compared to the others (R² = 0.211, β = −0.459, p < 0.001). Furthermore, when age was added, the explanatory power increased significantly in each of the models. For example, for BMI + age (R² = 0.435, β BMI = 0.22, β Age = −0.712, p = 0.000). For TWI + age (R² = 0.467, β TWI = 0.273, β age = −0.600, p = 0.000). And for BSA + age (R² = 0.394, β BSA = 0.052, β age = −0.667, p = 0.000). Finally, when sex was added, the explanatory power increased again in all three models. In the BMI + age + sex model (R² = 0.498, β BMI = 0.192, β Age = −0.681, β Sex = 0.254, p = 0.000). For the TWI + age + sex (R² = 0.517, β TWI= 0.229, β age= −0.586, β Sex= 0.228, p = 0.000). And for BSA + age (R² = 0.470, β BSA= 0.091, β age= −0.675, β Sex= −0.277, p = 0.000). Therefore, the model with TWI + age + sex showed the best performance in explaining the agility test (R² = 0.517, SEE = 2.760). Furthermore, the coefficient for age could be the most relevant predictor (β = −0.586). However, the TWI independently provides important information (β = 0.229), while sex has less significance (β = 0.228).

The percentiles for the velocity and agility test determined by the LMS method are shown in Table 4 for age and gender. In both cases, velocity and agility values decrease with age.

**Table 4:** Distribution of percentiles (LMS) for velocity and agility tests for schoolchildren of both sexes.

	Males														Females													
Age	L	M	S	P97	P95	P90	P85	P75	P50	P25	P15	P10	P5	P3	L	M	S	P97	P95	P90	P85	P75	P50	P25	P15	P10	P5	P3
	Velocity (s)														Velocity (s)													
6	−1.837	5.661	0.126	4.6	4.7	4.9	5.0	5.2	5.7	6.2	6.6	6.9	7.4	7.7	−1.222	5.639	0.115	4.7	4.8	4.9	5.0	5.2	5.6	6.1	6.4	6.6	7.0	7.2
7	−1.784	5.334	0.126	4.4	4.5	4.6	4.7	4.9	5.3	5.8	6.2	6.4	6.9	7.2	−1.346	5.368	0.118	4.4	4.5	4.7	4.8	5.0	5.4	5.8	6.1	6.4	6.7	7.0
8	−1.759	5.016	0.125	4.1	4.2	4.4	4.5	4.6	5.0	5.5	5.8	6.0	6.5	6.8	−1.436	5.135	0.121	4.2	4.3	4.5	4.6	4.8	5.1	5.6	5.9	6.1	6.5	6.8
9	−1.816	4.717	0.123	3.9	4.0	4.1	4.2	4.4	4.7	5.2	5.5	5.7	6.1	6.4	−1.464	4.954	0.122	4.1	4.2	4.3	4.4	4.6	5.0	5.4	5.7	5.9	6.3	6.6
10	−1.965	4.439	0.120	3.7	3.8	3.9	4.0	4.1	4.4	4.8	5.1	5.3	5.7	6.0	−1.419	4.806	0.124	3.9	4.0	4.2	4.3	4.4	4.8	5.3	5.5	5.8	6.1	6.4
11	−2.186	4.193	0.115	3.5	3.6	3.7	3.8	3.9	4.2	4.6	4.8	5.0	5.4	5.6	−1.319	4.687	0.126	3.8	3.9	4.0	4.2	4.3	4.7	5.1	5.4	5.6	6.0	6.2
12	−2.382	3.997	0.110	3.4	3.4	3.5	3.6	3.7	4.0	4.3	4.6	4.7	5.1	5.3	−1.176	4.570	0.129	3.7	3.8	3.9	4.0	4.2	4.6	5.0	5.3	5.5	5.8	6.1
13	−2.474	3.867	0.106	3.3	3.3	3.4	3.5	3.6	3.9	4.2	4.4	4.6	4.9	5.1	−1.003	4.482	0.133	3.6	3.7	3.8	3.9	4.1	4.5	4.9	5.2	5.4	5.7	6.0
14	−2.441	3.793	0.103	3.2	3.3	3.4	3.4	3.6	3.8	4.1	4.3	4.4	4.7	4.9	−0.828	4.461	0.138	3.5	3.6	3.8	3.9	4.1	4.5	4.9	5.2	5.4	5.7	6.0
15	−2.319	3.791	0.102	3.2	3.3	3.4	3.5	3.6	3.8	4.1	4.3	4.4	4.7	4.9	−0.683	4.508	0.143	3.5	3.6	3.8	3.9	4.1	4.5	5.0	5.3	5.5	5.8	6.1
16	−2.151	3.799	0.101	3.2	3.3	3.4	3.5	3.6	3.8	4.1	4.3	4.4	4.7	4.8	−0.582	4.574	0.147	3.5	3.6	3.8	4.0	4.2	4.6	5.1	5.4	5.6	5.9	6.2
17	−1.956	3.782	0.100	3.2	3.3	3.4	3.4	3.5	3.8	4.1	4.2	4.4	4.6	4.8	−0.523	4.641	0.152	3.6	3.7	3.9	4.0	4.2	4.6	5.2	5.5	5.7	6.1	6.3
	Agility 5 × 10 m (s)														Agility 5 × 10 m (s)													
6	−0.883	26.793	0.158	20.6	21.2	22.2	23.0	24.2	26.8	30.0	32.0	33.5	36.0	37.8	−0.482	26.952	0.163	20.3	21.0	22.1	22.9	24.2	27.0	30.2	32.1	33.6	35.9	37.5
7	−0.797	25.192	0.149	19.6	20.1	21.1	21.8	22.9	25.2	28.0	29.7	31.0	33.1	34.6	−0.439	25.586	0.158	19.4	20.0	21.1	21.8	23.1	25.6	28.5	30.3	31.6	33.7	35.2
8	−0.648	23.639	0.140	18.5	19.1	19.9	20.6	21.6	23.6	26.1	27.5	28.6	30.4	31.6	−0.407	24.281	0.152	18.5	19.1	20.1	20.8	22.0	24.3	27.0	28.6	29.8	31.6	32.9
9	−0.421	22.183	0.133	17.5	18.0	18.8	19.4	20.3	22.2	24.3	25.6	26.5	27.9	28.9	−0.405	23.077	0.147	17.8	18.3	19.3	19.9	20.9	23.1	25.5	27.0	28.1	29.7	30.9
10	−0.122	20.848	0.126	16.5	17.0	17.8	18.3	19.2	20.8	22.7	23.8	24.5	25.7	26.5	−0.417	21.967	0.140	17.1	17.6	18.5	19.1	20.0	22.0	24.2	25.5	26.5	28.0	29.0
11	0.177	19.648	0.121	15.6	16.1	16.8	17.3	18.1	19.6	21.3	22.2	22.9	23.9	24.5	−0.462	21.192	0.134	16.7	17.2	18.0	18.5	19.4	21.2	23.2	24.5	25.4	26.8	27.7
12	0.360	18.677	0.116	14.9	15.3	16.0	16.5	17.2	18.7	20.2	21.0	21.6	22.5	23.0	−0.524	20.707	0.130	16.5	16.9	17.6	18.2	19.0	20.7	22.7	23.8	24.7	26.0	26.9
13	0.371	18.036	0.112	14.5	14.9	15.6	16.0	16.7	18.0	19.4	20.2	20.7	21.6	22.1	−0.536	20.372	0.128	16.3	16.7	17.4	17.9	18.7	20.4	22.2	23.4	24.2	25.5	26.3
14	0.215	17.598	0.110	14.3	14.6	15.3	15.7	16.3	17.6	18.9	19.7	20.2	21.0	21.5	−0.461	20.254	0.126	16.2	16.6	17.3	17.8	18.6	20.3	22.1	23.2	24.0	25.2	26.1
15	−0.117	17.484	0.108	14.3	14.7	15.2	15.6	16.3	17.5	18.8	19.6	20.1	20.9	21.5	−0.265	20.236	0.126	16.1	16.5	17.3	17.8	18.6	20.2	22.1	23.1	23.9	25.0	25.8
16	−0.579	17.461	0.108	14.4	14.7	15.3	15.7	16.3	17.5	18.8	19.6	20.2	21.1	21.7	−0.005	20.254	0.125	16.0	16.5	17.2	17.8	18.6	20.3	22.0	23.1	23.8	24.9	25.6
17	−1.113	17.470	0.108	14.5	14.9	15.4	15.7	16.3	17.5	18.9	19.7	20.3	21.3	22.0	0.262	20.285	0.125	15.9	16.4	17.2	17.8	18.6	20.3	22.0	23.0	23.7	24.8	25.5

## Discussion

The main objective of this study was to verify the applicability of anthropometric indices (BMI, TWI, and BSA) for evaluating velocity and agility physical tests in Chilean children and adolescents. The results of the study showed that the TWI exhibited more consistent behavior in the regression models for both physical tests (velocity and agility). These findings suggest that the TWI may better represent the morphological characteristics associated with motor performance during childhood and adolescence.

These results are similar to other studies that have highlighted that the TWI may be a better predictor for estimating physical fitness, motor competence, body adiposity, and metabolic risk in children and adolescents [6,19].

In fact, a study conducted >9 years ago suggested that TWI is a more stable anthropometric indicator than BMI in children and adolescents [20]. Since it considers the relationship between body weight and height cubed, it allows for a more accurate representation of body proportionality during growth. Furthermore, height cubed helps correct for variations not only due to height but also due to age and weight, especially during the growth pase [6]. However, BSA primarily reflects total body dimensions (body size). Therefore, it is more influenced by changes associated with growth and maturation [21].

These characteristics could explain why, in our results, TWI showed more consistent associations with velocity and agility once the effects of age and sex were controlled for, compared to BSA and BMI, respectively.

Therefore, TWI could better discriminate variations in body composition. Identifying both increases in fat mass in overweight or obese children and differences in fat-free mass in leaner children [22]. However, further studies including direct measurements of body composition, such as dual-energy X-ray absorptiometry (DXA), are needed to confirm these potential relationships with motor performance in children and adolescents.

In practical terms, our results suggest that the TWI could be a more sensitive tool than the BSA and BMI for studying the relationship between body morphology and motor performance in schoolchildren, especially when assessing neuromuscular abilities such as veocity and agility.

The second objective of the study was to develop percentiles for velocity and agility for Chilean schoolchildren according to age and sex. To this end, this study used the Lambda-Mu-Sigma (LMS) method. The three curves can be fitted as cubic splines using nonlinear regression, and the degree of smoothing required can be expressed in terms of smoothing parameters or equivalent degrees of freedom [23].

This technique has been widely used by several studies to propose physical performance percentiles in schoolchildren in various regions of the world [24,25]. This demonstrates the uses and applications of the LMS method, which allows the performance of a child to be visualized over time [26]. In addition, they can serve as a tool to be implemented in schools [27] and to monitor and guide schoolchildren who need interventions [28].

The cutoff points considered in this study are described in three categories, where children scoring below the 15th percentile (<p15) are characterized by warning signs of underperformance (functional risk). Meanwhile, those scoring between p15 and p85 are considered adequate. Those between p15 and p85 are considered adequate, and those above p85 are considered to have a high level of physical performance [24,29].

The study’s percentile thresholds (P15 and P85) were selected based on previous pediatric reference studies. They should be interpreted as practical normative categories rather than cutoff values for clinical diagnosis. In a school setting, these thresholds can help classify children with comparatively lower or higher physical performance according to their age and sex. However, the suitability of these percentile limits for identifying functional limitations or athletic potential requires further validation studies. This could be done using external criteria, such as motor competence, sports participation, or health-related outcomes.

In summary, the percentiles developed for agility and velocity allow physical performance levels to be determined and identified. For example, velocity is a complex, multiphase motor skill that involves coordination, application of force, and control of stiffness [30]. Agility is the motor skill that involves performing a set of movements that allow the body to change direction and position quickly and accurately, without losing balance or awareness of body position.

Therefore, the assessment of both indicators of the motor component of physical fitness in schoolchildren is relevant in terms of motor skills and athletic performance. It is widely applicable to educational contexts for the evaluation, monitoring, and planning of physical education classes and sports initiation programs.

### Practical implications

**Figure 2:**
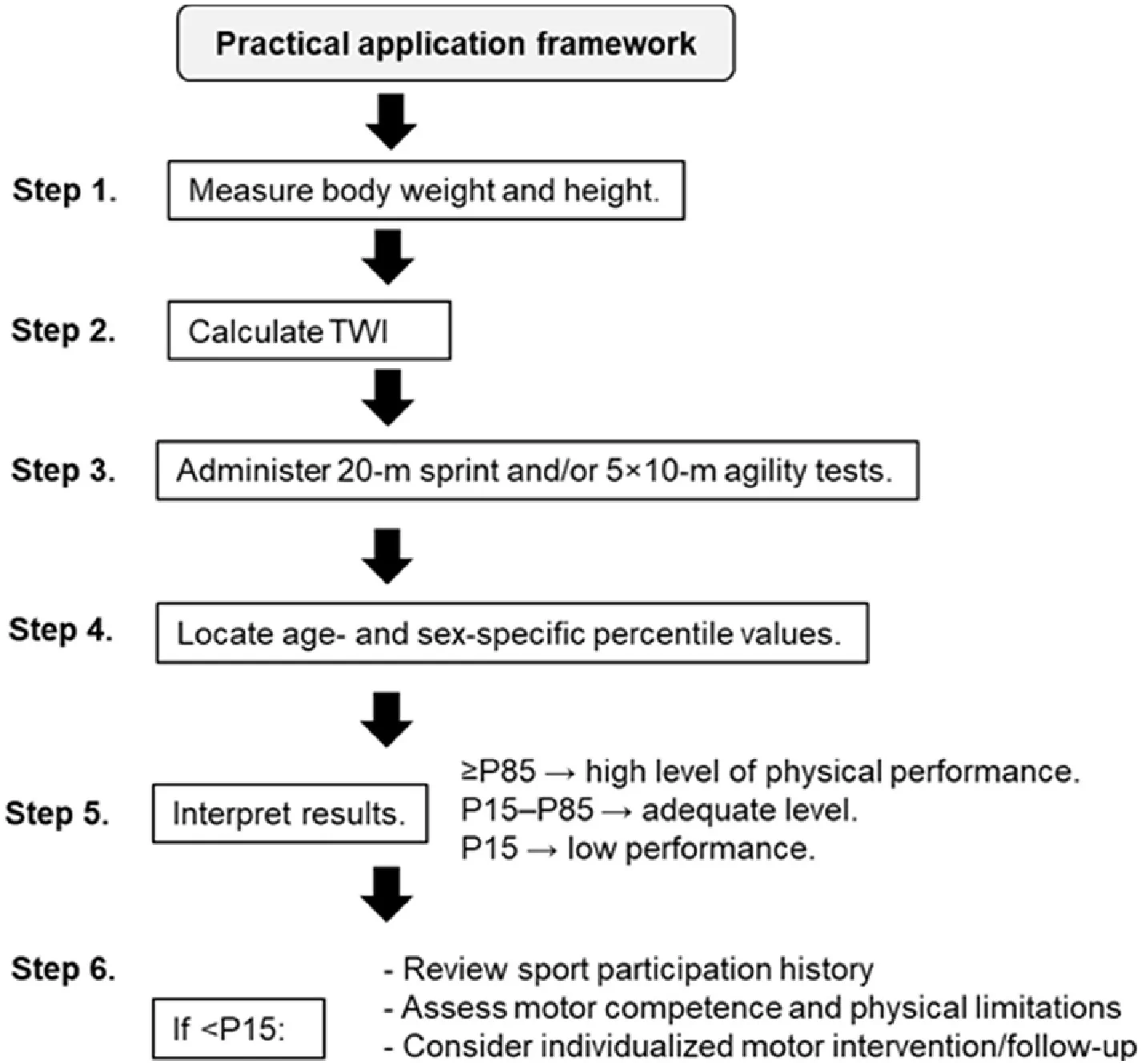
Algorithm on the practical use of anthropometric indices in the evaluation of velocity and agility in schoolchildren.

From a practical standpoint, educational institutions and pediatric health centers can use routinely collected anthropometric data (weight and height) to calculate the TWI. Then, after conducting 20-meter sprint and/or 5 × 10-meter agility tests, they can interpret the results using age- and sex-specific percentile references. Figure 2 illustrates this practical application, which would involve identifying children performing below the 15th percentile. In cases of low performance, it is suggested that a supplementary motor skills assessment be conducted, any physical limitations be identified, or the child be considered for inclusion in an intervention program and longitudinal follow-up.

The study has some strengths: it is one of the first studies to investigate these two motor tests transversally across a wide age range and in both sexes. In addition, these results can serve as a baseline for future research, making it possible to study changes in the secular trends of these variables.

Furthermore, practical implications include the fact that schools can use weight and height to calculate the TWI (tri- weight index). They can then place children into speed/agility percentiles (Table 4) for longitudinal monitoring and functional assessment.

In addition, percentiles can help teachers and clinical centers interpret performance in relation to age and gender.

The study also has some limitations, particularly related to the type of study, since a cross-sectional design was used and future studies need to develop longitudinal studies. This opens up possibilities for verifying and testing causal relationships. In addition, the sample selection was non-probabilistic, which prevents the results from being generalized to other sociocultural contexts.

It is also important to consider the assessment of biological maturation when analyzing physical performance in children and adolescents. This involves verifying the behavior of peak growth velocity years (APHV) as an additional covariate to generate complementary models. However, in this study, after controlling for maturity status using anthropometric methods (APHV), we observed severe multicollinearity in the generated regression models. This prevented its inclusion in the results. Nevertheless, we have included a supplementary table 1 where these results can be seen.

Also the use of stopwatches in the physical tests in this study could show some biases in the results. Therefore, future studies could use sophisticated technologies (photocells) to improve the reliability of the results. Studies should also calculate the sample size according to the sociodemographic characteristics of Chilean schoolchildren.

The results show that the TWI had a greater explanatory capacity for motor performance compared to BMI and BSA in Chilean schoolchildren. Models that incorporated TWI along with age and sex showed the highest coefficients of determination, suggesting that this indicator could be an alternative for assessing velocity and agility. Furthermore, percentiles were developed for the 20-meter sprint and 5 × 10-meter agility tests, allowing for the monitoring, interpretation, and verification of changes in motor performance in school settings.

## Conflicts of interest

The authors declare no conflicts of interest.

## Data Availability

The data that support the findings of this study are available from the corresponding author.

## Appendix Supplementary materials

Image, application 1

## References

[1] Casadei K., Kiel J. (2026). StatPearls [Internet].

[2] Mates E., Lelijveld N., Ali Z., Sadler K., Yarparvar A., Walters T. (2023). Nutrition of school-aged children and adolescents in Europe and Central Asia Region: a literature and survey review. Food Nutr Bull.

[3] Cossio-Bolaños M., Vidal Espinoza R., Sulla-Torres J., Urra-Albornoz C., Sanchez-Macedo L., de Arruda M. (2025). validation of body surface area equations for estimating fat-free mass by dual x-ray absorptiometry in a regional Chilean sample aged 4 to 85 years. Diagn (Basel).

[4] Stanković M., Đorđević D., Zelenović M., Božić D. (2021). Correlation of body composition with speed and agility of children aged 9-10. Ann Kinesiol.

[5] Pezoa-Fuentes P., Cossio-Bolaños M., Urra-Albornoz C., Alvear-Vasquez F., Lazari E., Urzua-Alul L. (2023). Fat-free mass and maturity status are determinants of physical fitness performance in schoolchildren and adolescents. J Pediatr (Rio J).

[6] Cossio-Bolaños M., Vidal-Espinoza R., Albornoz C.U., Fuentes-Lopez J., Sánchez-Macedo L., Andruske C.L. (2022). Relationship between the body mass index and the ponderal index with physical fitness in adolescent students. BMC Pediatr.

[7] Fuentes-Lopez J., Vidal-Espinoza R., Silva D.R., Mamani-Velásquez D., Vargas-Ramos E., Pacompia-Cari E. (2026). Applicability of anthropometric indicators to assess physical fitness: proposal of percentiles for schoolchildren living at high altitude in Peru. J Pediatr (Rio J).

[8] Köklü Y., Alemdaroğlu U., Özkan A., Koz M., Ersöz G. (2015). The relationship between sprint ability, agility and vertical jump performance in young soccer players. Sci Sports.

[9] Di Domenico F., D’isanto T. (2019). Role of speed and agility in the effectiveness of motor performance. J Phys Educ Sport.

[10] Ross A., Leveritt M., Riek S. (2001). Neural influences on sprint running: training adaptations and acute responses. Sports Med.

[11] Sheppard J.M., Young W.B. (2006). Agility literature review: classifications, training and testing. J Sports Sci.

[12] Ross W.D, Marfell-Jones M.J (1991). Physiological testing of elite athlete. Hum Kinet.

[13] Du Bois D. (1916). Clinical calorimetry: tenth paper a formula to estimate the approximate surface area if height and weight be known. Arch Intern Med (Chic Ill: 1908).

[14] Moore S.A., McKay H.A., Macdonald H., Nettlefold L., Baxter-Jones A.D., Cameron N. (2015). Enhancing a somatic maturity prediction model. Med Sci Sports Exerc.

[15] Grosser M., Starischka S. (1988).

[16] Verschuren O., Takken T., Ketelaar M., Gorter J.W., Helders P.J. (2007). Reliability for running tests for measuring agility and anaerobic muscle power in children and adolescents with cerebral palsy. Pediatr Phys Ther.

[17] Pan H., Cole T.J. (2006). LMS chartmaker. http://www.healthforallchildren.co.uk.

[18] Cole T.J., Bellizzi M.C., Flegal K.M., Dietz W.H. (2000). Establishing a standard definition for child overweight and obesity worldwide: international survey. BMJ.

[19] Carrasco-López S., Valenzuela-Jiménez A., Amigo Alvear V., Irribarra Sepúlveda F., Baeza Parra F., Herrera Placencia C. (2024). Aplicabilidad del índice de masa corporal e índice tri-ponderal para valorar la competencia motora en escolares. Sport Sci J.

[20] Peterson C.M., Su H., Thomas D.M., Heo M., Golnabi A.H., Pietrobelli A. (2017). Tri-ponderal mass index vs body mass index in estimating body fat during adolescence. JAMA Pediatr.

[21] Alvear-Vásquez F., Vidal-Espinoza R., Gomez-Campos R., de Campos L.F., Lazari E., Guzmán-Luján J.F. (2023). Body surface area is a predictor of maturity status in school children and adolescents. BMC Pediatr.

[22] Moselakgomo V.K., Van Staden M. (2019). Diagnostic accuracy of tri-ponderal mass index and body mass index in estimating overweight and obesity in South African children. Afr J Prim Health Care Fam Med.

[23] Cole T.J., Green P.J. (1992). Smoothing reference centile curves: the LMS method and penalized likelihood. Stat Med.

[24] Hobold E., Pires-Lopes V., Gómez-Campos R., de Arruda M., Andruske C.L., Pacheco-Carrillo J. (2017). Reference standards to assess physical fitness of children and adolescents of Brazil: an approach to the students of the Lake Itaipú region-Brazil. PeerJ.

[25] Yang Y., Hadier S.G., Long L., Hamdani S.M., Hamdani S.D. (2025). Development and cross-validation of LMS-based normative reference standards and health benefits zones for muscular strength among adolescents by age and sex. Front Public Health.

[26] Ulrich S., Hildenbrand F.F., Treder U., Fischler M., Keusch S., Speich R. (2013). Reference values for the 6-minute walk test in healthy children and adolescents in Switzerland. BMC Pulm Med.

[27] Sulla-Torres J., Vidal-Espinoza R., Avendaño-Llanque C., Calla-Gamboa A., Zúñiga-Carnero M., Gomez-Campos R. (2024). Reference values for the 6-min walking test in children and adolescents living in a moderate altitude region of Peru. BMC Pediatr.

[28] Meng Y., Song Y., Li H. (2025). Cardiorespiratory fitness in Chinese children and adolescents: a systematic review and meta-analysis. Ann Hum Biol.

[29] Gómez-Campos R., Andruske C.L., Arruda M., Sulla-Torres J., Pacheco-Carrillo J., Urra-Albornoz C. (2018). Normative data for handgrip strength in children and adolescents in the Maule Region, Chile: evaluation based on chronological and biological age. PLoS One.

[30] Bleeker S., Siener M. (2025). Development of juvenile sprint performance in boys: analysis of speed phases-a cross-sectional study by age. Front Sports Act Living.

