# Supplementary material for: Applicability of anthropometric indices for assessing speed and agility in children and adolescents: proposed reference values by age and sex
Source: J Pediatr (Rio J). 2026 Sep 5;102(6):101596. doi: 10.1016/j.jped.2026.101596 (PMC13571986; doi:10.1016/j.jped.2026.101596)
Supplement: Supplementary file 1 [file mmc1.docx]

**JPED-D-26-00058_ Supplementary Material**

**Supplementary Table 1**

|  | **R** | **R²** | **Adj R²** | **SEE** |  | | **p** | **Tol** | **VIF** | **R** | **R²** | **Adj R²** | **SEE** |  | | **p** | **Tol** | **VIF** |
| --- | --- | --- | --- | --- | --- | --- | --- | --- | --- | --- | --- | --- | --- | --- | --- | --- | --- | --- |
| **Velocity** | | | | | | | | | | **Agility** | | | | | | | | |
| BMI + Age + Sex + APHV | .640 | 0.409 | 0.408 | 0.666 | Cte |  | 0.000 |  |  | .706 | 0.498 | 0.497 | 2.814 | Cte |  | 0.000 |  |  |
|  |  |  |  |  | β BMI | 0.217 | 0.000 | 0.730 | 1.370 |  |  |  |  | β BMI | 0.190 | 0.000 | 0.735 | 1.361 |
|  |  |  |  |  | β Age | -0.599 | 0.000 | 0.070 | 14.346 |  |  |  |  | β Age | -0.702 | 0.000 | 0.070 | 14.350 |
|  |  |  |  |  | β Sex | 0.263 | 0.000 | 0.215 | 4.654 |  |  |  |  | β Sex | 0.243 | 0.000 | 0.210 | 4.762 |
|  |  |  |  |  | β APHV | -0.001 | 0.987 | 0.056 | 17.953 |  |  |  |  | β APHV | 0.024 | 0,738 | 0.055 | 18.224 |
| TWI + Age + Sex + APHV | .660 | 0.435 | 0.434 | 0.651 | Cte |  | 0.000 |  |  | .719 | 0.517 | 0.516 | 2.760 | Cte |  | 0.000 |  |  |
|  |  |  |  |  | β TWI | 0.260 | 0.000 | 0.890 | 1.124 |  |  |  |  | β TWI | 0.227 | 0.000 | 0.889 | 1.125 |
|  |  |  |  |  | β Age | -0.503 | 0.000 | 0.068 | 14.689 |  |  |  |  | β Age | -0.622 | 0.000 | 0.068 | 14.658 |
|  |  |  |  |  | β Sex | 0.231 | 0.000 | 0.227 | 4.399 |  |  |  |  | β Sex | 0.210 | 0.000 | 0.222 | 4.498 |
|  |  |  |  |  | β APHV | 0.011 | 0.885 | 0.059 | 16.839 |  |  |  |  | β APHV | 0.041 | 0,551 | 0.059 | 17.076 |
| BSA + Age + Sex + APHV | .613 | 0.375 | 0.374 | 0.685 | Cte |  | 0.000 |  |  | .687 | 0.472 | 0.471 | 2.885 | Cte |  | 0.000 |  |  |
|  |  |  |  |  | β BSA | 0.042 | 0.222 | 0.325 | 3.076 |  |  |  |  | β BSA | 0.054 | 0.075 | 0.328 | 3.049 |
|  |  |  |  |  | β Age | -0.757 | 0.000 | 0.073 | 13.690 |  |  |  |  | β Age | -0.834 | 0.000 | 0.073 | 13.729 |
|  |  |  |  |  | β Sex | 0.175 | 0.000 | 0.190 | 5.259 |  |  |  |  | β Sex | 0.177 | 0.000 | 0.184 | 5.433 |
|  |  |  |  |  | β APHV | 0.236 | 0.006 | 0.052 | 19.398 |  |  |  |  | β APHV | 0.211 | 0.006 | 0.051 | 19.799 |
